# Transferrin receptor-1 and VEGF are prognostic factors for osteosarcoma

**DOI:** 10.1186/s13018-019-1301-z

**Published:** 2019-09-04

**Authors:** Hongzeng Wu, Jinming Zhang, Ruoheng Dai, Jianfa Xu, Helin Feng

**Affiliations:** 1 Department of Orthopedics, The Fourth Hospital of Hebei Medical University, 12 Health Road, Shijiazhuang, 050011 Hebei People’s Republic of China; 2 Department of Pediatrics, The Fourth Hospital of Hebei Medical University, 12 Health Road, Shijiazhuang, 050011 Hebei People’s Republic of China

**Keywords:** Transferrin receptor-1, VEGF, Prognosis, Osteosarcoma

## Abstract

**Background:**

Osteosarcoma is aggressive and prognostic biomarkers are important to predict the outcomes of surgery and chemotherapy. Here, we investigated the potential of transferrin receptor-1 (TfR1) and vascular endothelial growth factor (VEGF) as prognostic markers of osteosarcoma.

**Methods:**

TfR1 and VEGF in osteosarcoma samples from a cohort of 53 osteosarcoma patients were detected by immunohistochemistry analysis. The correlation of TfR1 and VEGF levels with clinicopathological parameters was analyzed by Pearson chi-square and Spearman-rho tests. Overall patient survival was analyzed by the Kaplan-Meier method.

**Results:**

We found that TfR1 and VEGF expression levels were low in 20.8% and 18.9%; modest in 35.8% and 35.8%; and high in 43.4% and 45.3% of osteosarcoma patients, respectively. TfR1 and VEGF expression was significantly correlated to histologic grade, Enneking stage, and distant metastasis. TfR1 expression was significantly correlated to VEGF expression and both TfR1 expression and VEGF expression were correlated to shorter overall survival.

**Conclusions:**

TfR1 and VEGF are potential prognostic factors for osteosarcoma.

## Background

Primary bone tumors are uncommon and the incidence is low [1]. Osteosarcoma (OS) is a pleomorphic sarcoma of the bone in children and adult, and OS patients frequently develop metastasis [2]. With the recent development of adjuvant chemotherapy, the 5-year-free survival rate has improved to approximately 50% for patient with high-grade OS [3, 4]. The identification of new prognostic biomarkers in osteosarcoma has become increasingly important to predict the responsiveness of treatment [5].

Iron is an element essential to cellular activities such as DNA synthesis and cell proliferation [6–8]. Proteins involved in iron metabolism have been shown to promote lung cancer [9–11]. Recent studies have shown high expression of transferrin receptor-1 (TfR1) in a variety of tumors including lung, breast, and bladder cancer as well as malignant glioma, but the clinical significance of TfR1 in tumor remains to be confirmed [12, 13].

Angiogenesis plays an important role in tumor development. Vascular endothelial growth factor (VEGF) is known to promote neovascularization [14, 15]. Up to now, the association between TfR1 and VEGF expression and the prognosis of OS patients remains unclear. Therefore, this study aimed to examine TfR1 and VEGF expression in OS patients and analyze their prognostic significance for clinical outcomes of OS.

## Methods

### Subjects

Ethics Committees of the Fourth Hospital of Hebei Medical University (also named as Tumor Hospital of Hebei Province) approved this study and all patients signed written informed consent. This study enrolled 53 OS patients from 2002 to 2010 from the Fourth Hospital of Hebei Medical University, who had not received radiotherapy or chemotherapy. All patient data and follow-up information were collected, including the gender, age, tumor size, histological grade, Enneking stage, and distant metastasis.

### Immunohistochemistry analysis

Immunohistochemistry (IHC) analysis was performed on OS tissues using antibodies for TfR1 (1:100; Biogot Tech) and VEGF (1:100; Santa Cruz Biotechnology), following a previously described protocol [16]. The results of IHC were judged using the following score system based on the percentage of stained cells, < 1% (0); 1–25% (1); 25–50% (2); 51–80% (3); and > 80% (4); and the intensity of staining, no staining (0); weak staining (1); strong staining (2); and very strong staining (3). The final score was the product of staining intensity and percentage and judged as low (0–3 points), mild (4–7 points), and high (> 7 points).

### Statistical analysis

All data were analyzed by using SPSS software 25.0. The association of clinical variables was analyzed by the Pearson chi-square test or Spearman-rho test. Univariate and multivariate analyses were performed by using the Cox proportional hazard model. Survival was analyzed by the Kaplan-Meier method. *P* < 0.05 was considered significant.

## Results

### Association of TfR1 and VEGF with clinicopathological parameters

**Fig. 1:**
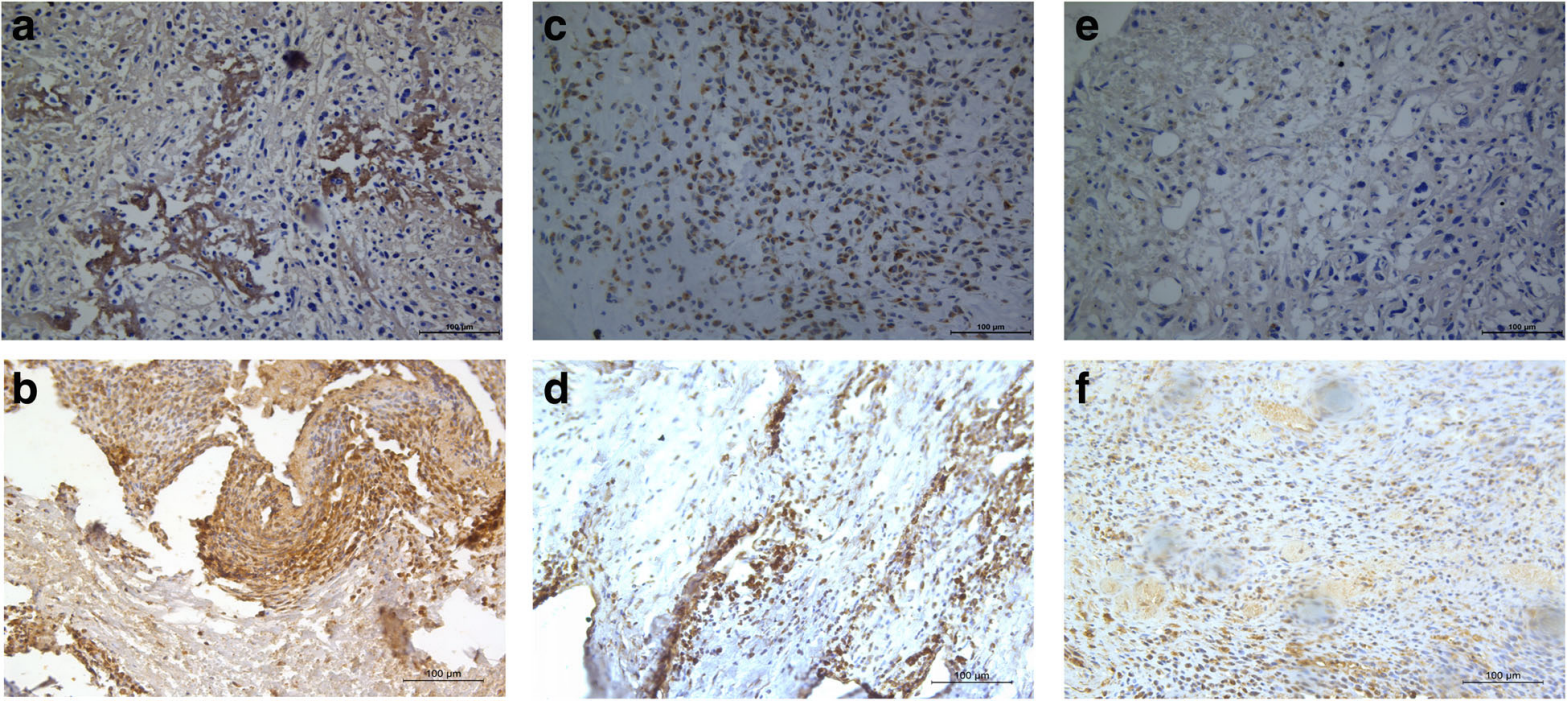
Representative immunohistochemical staining of TfR1 and VEGF. **a** High expression of TfR1 in OS. **c** Moderate expression of TfR1 in OS. **e** Low expression of TfR1 in OS. **b** High expression of VEGF in OS. **d** Moderate expression of VEGF in OS. **f** Low expression of VEGF in OS. The cells with positive expression were stained brown

Typical staining of TfR1 and VEGF in OS tissues was presented in Fig. 1. TfR1 expression was low in 20.8%, mild in 35.8% and high in 43.4% of OS tissues, whereas VEGF expression was low in 18.9%, mild in 35.8%, and high in 45.3% of OS tissues. As shown in Table 1, TfR1 and VEGF expression was significantly associated with histological grade, Enneking stage and distant metastasis (all *P* < 0.05). In addition, TfR1 and VEGF expression showed a significantly positive correlation (*P* < 0.01, Table 2).

**Table 1:** Clinicopathological variables and the expression of TfR1 and VEGF

			TfR1			*P*	VEGF			*P*
			Low(%)	Mild(%)	High(%)		Low(%)	Mild(%)	High(%)	
Sex	Female	25	3(12.0)	10(40.0)	12(48.0)	0.332	5(20.0)	11(44.0)	9(36.0)	0.405
	Male	28	8(28.6)	9(32.1)	11(39.3)		5(17.8)	8(28.6)	15(53.6)	
Age	≥ 20 years	18	4(22.2)	4(22.2)	10(55.6)	0.306	3(16.7)	9(50.0)	6(33.3)	0.293
	< 20 years	35	7(20.0)	15(42.9)	13(37.1)		7(20.0)	10(28.6)	18(51.4)	
Tumor size	< 5 cm	27	6(22.2)	10(37.0)	11(40.8)	0.919	6(22.2)	10(37.0)	11(40.7)	0.741
	≥ 5 cm	26	5(19.2)	9(34.6)	12(46.2)		4(15.4)	9(34.6)	13(50.0)	
Histologic grade*	I	15	4(26.7)	8(53.3)	3(20.0)	0.04	4(26.7)	9(60.0)	2(13.3)	0.02
	II	25	5(20.0)	10(40.0)	10(40.0)		5(20.0)	8(32.0)	12(48.0)	
	III	13	2(15.4)	1(7.7)	10(76.9)		1(7.7)	2(15.4)	10(76.9)	
Distant metastasis*	Yes	23	1(4.2)	11(47.8)	11(47.8)	0.029	1(4.4)	13(56.5)	9(39.1)	0.008
	No	30	10(33.3)	8(26.7)	12(40.0)		9(30.0)	6(20.0)	15(50.0)	
Enneking staging*	I	12	8 (66.7)	3 (25.0)	7(36.9)	< 0.001	6(50.0)	4(33.3)	2(16.7)	0.004
	II	19	2 (10.5)	10(52.6)	21(43.8%)		2(10.5)	10(52.6)	7(36.9)	
	III	22	1 (4.5)	6 (27.3)	15 (68.2)		2(9.1)	5(22.7)	15(68.2)	

Pearson’s chi-squared test was used. *P < 0.05

**Table 2:** The correlation of TfR1 and VEGF expression

Characteristics			TfR1			*P* (Spearman)
			Low(%)	Mild(%)	High(%)	
VEGF*	Low	10	6(11.3)	3(5.7)	1(1.9)	= 0.001
	Mild	19	2(3.7)	10(18.9)	7(13.2)	
	High	24	3(5.7)	6(11.3)	15(28.3)	
		53	11	19	23	

Spearman-rho test was used. *P < 0.05

### TfR1 and VEGF were correlated with poor overall survival of OS patients

**Fig. 2:**
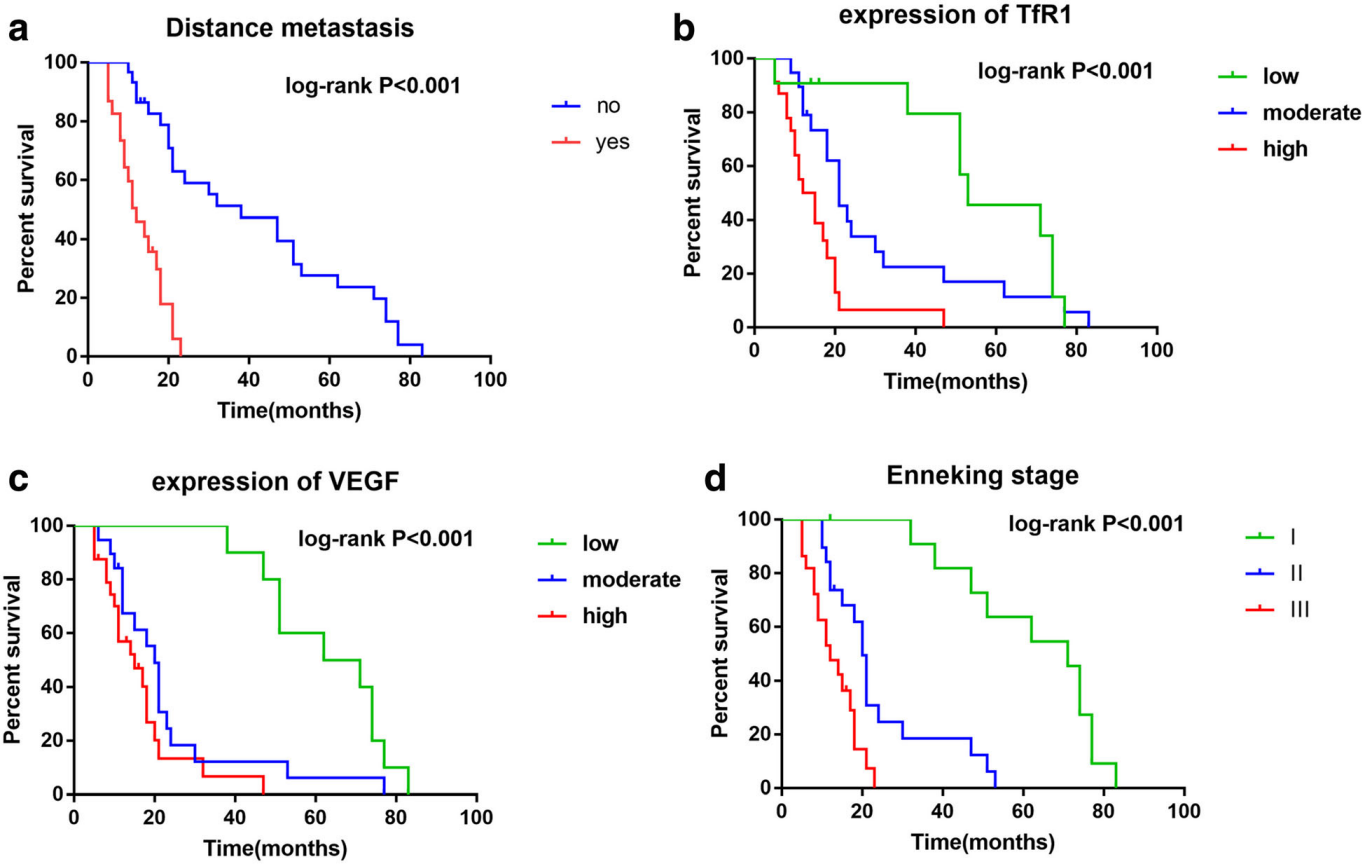
Overall survival curves of patients with OS. **a** Association of overall survival with distant metastasis. **b** Association of overall survival with TfR1 expression. **c** Association of overall survival with VEGF expression. **d** Association of overall survival with clinical stage

Table 3 showed the results of univariate Cox hazard analysis of overall survival of OS patients. Kaplan-Meier survival curve showed that the gender, age, tumor size, and histologic grade had no significance in predicting overall survival, but Enneking staging and distant metastasis predicted a poor overall survival (Fig. 2). Moreover, TfR1 and VEGF were significantly correlated with poor overall survival (Table 3, Fig. 2).

**Table 3:** Clinicopathological factors associated with overall survival based on univariate Cox proportional regression analysis

Characteristics			Overall survival		*P*
			HR	95% CI	
Sex	Female	25	1		0.837
	Male	28	1.064	0.590–1.919	
Age	≥ 20 years	18	1		0.777
	< 20 years	35	1.093	0.591–2.022	
Tumor size	< 5 cm	27	1		0.940
	≥ 5 cm	26	1.024	0.556–1.884	
Histologic grade*	I	15	1		0.412
	II	25	1.267	0.634–2.534	0.503
	III	13	1.738	0.771–3.917	0.183
Distant metastasis*	Yes	23	1		< 0.001
	No	30	0.161	0.073–0.356	
Enneking staging*	I	12	1		< 0.001
	II	19	8.605	2.942–25.169	
	III	22	26.039	7.679–88.293	
TfR1*	Low	11	1		< 0.001
	Moderate	19	0.158	0.063–0.398	
	High	23	0.300	0.143–0.629	
VEGF*	Low	10	1		0.021
	Moderate	19	0.114	0.043–0.303	
	High	24	0.422	0.202–0.880	

*HR*, hazard ratio; *95% CI*, 95% confidence interval. *P < 0.05

### TfR1 and VEGF are prognostic factors for OS patients

Table 4 showed the results of multivariate Cox hazard analysis of univariate factors listed in Table 3. Enneking staging, TfR1 expression, and VEGF expression were identified as independent prognostic factors of OS patients. Higher TfR1 and VEGF expression, higher Enneking staging, and distance metastasis were associated with significantly higher mortality risk (Plogrank < 0.001) (Fig. 2).

**Table 4:** Clinicopathological factors associated with overall survival based on multivariate Cox regression analysis

	Overall survival		
	HR	95% CI	*P*
Enneking stage	4.622	2.541–8.406	< 0.001
TfR1	2.514	1.445–4.372	0.001
VEGF	2.882	1.203–8.217	0.002

*HR*, hazard ratio; *95% CI*, 95% confidence interval. *P < 0.05

## Discussion

As a common malignant bone tumor, OS accounts for 30% of all bone malignancies and 3–4% of pediatric tumors [17]. OS has been reported to be the third most common cancer in adolescence [18]. Therefore, it is important to identify novel biomarkers and therapeutic targets for OS.

Abnormal iron metabolism is associated with tumorigenesis [19–21]. Iron homeostasis is maintained by the balance of iron uptake, usage, and storage [22]. TfR1 is the main protein responsible for iron absorption. Strong immunohistochemical staining of TfR1 could indicate high cancer cell proliferation and poor prognosis of cancer patients [23–25]. Tumor cells with high TfR1 expression exhibited a high rate of iron absorption and cell proliferation [26].

To our knowledge, our study was the first to report high expression of TfR1 and VEGF in OS tissues. Moreover, we found that high TfR1 and VEGF expression was significantly correlated to histological grade, Enneking staging, and distant metastasis. Furthermore, high TfR1 and VEGF expression was significantly correlated to poor overall survival, and both TfR1 and VEGF were independent prognostic indicators of OS patients.

Our study has several limitations. First, immunohistochemistry analysis is only semi-quantitative, and bias may affect the evaluation of staining score although we analyzed all samples in a blind manner. Second, our sample size is limited. Third, our study is a single-center study.

## Conclusions

In summary, TfR1 and VEGF expression is high in OS tissues and is correlated to malignancy grade of OS patients. TfR1 and VEGF are potential prognostic factors of OS patients.

## Data Availability

All data and material are available upon request

## Abbreviations

OS: Osteosarcoma
TfR1: Transferrin receptor-1
VEGF: Vascular endothelial growth factor
